# Editorial for Special Issue: “Preparation of Nanomaterial Modified Electrode and Its Sensing Application”

**DOI:** 10.3390/nano12224010

**Published:** 2022-11-15

**Authors:** Dong Liu, Baiqing Yuan

**Affiliations:** 1 School of Agricultural Engineering, Jiangsu University, Zhenjiang 212013, China; 2 School of Chemistry and Materials Science, Ludong University, Yantai 264025, China

Electrochemical sensors have attracted enormous attention for their precision, high sensitivity, rapid response, and ease-of-use for analysis. Electrochemical sensors use an electrode as transducer element for the target. In particular, nanomaterials with excellent properties are essential for the modification of electrodes, which highlights the importance of research on the preparation of nanomaterials for electrochemical sensing. Our Special Issue includes eleven research papers, consisting of nine articles and two reviews. These reports cover the preparation of nanomaterials (magnesium phyllosilicate [1], metal–organic frameworks (MOF) [2], and covalent–organic frameworks (COF) [3]), the fabrication of electrodes with unique properties (e.g., transparency) using carbon nanomaterials [4,5,6,7] or new nanotechnologies [8,9], and applications of electrochemical sensors [10,11].

Pecheu et al. synthesized a talc-like magnesium phyllosilicate functionalized with amine groups (TalcNH_2_) via a sol–gel process to fabricate electrochemical sensors for stripping voltametric analysis. The TalcNH_2_-modified electrode was used for the detection of Pb^2+^ with anodic adsorptive stripping differential pulse voltammetry. The detection and quantification limits were calculated to be 7.45 × 10^−8^ M and 24.84 × 10^−8^ M, respectively, for TalcNH_2_-based sensors [1]. This work provided an attracting nanomaterial for the fabrication of electrochemical sensors. Zhang et al. used the Co–Cu MOF precursor to synthesize porous spindle-like Cu–Co sulfide microparticles via solvothermal sulfurization. The resultant Cu–Co sulfides exhibited high catalytic activity for the electrochemical oxidation of glucose. The Co–CuS-2-based sensor exhibited enhanced analytical performance for the nonenzymatic electrochemical detection of glucose, which allowed a sensitivity of 1475.97 A mM^−1^ cm^−2^ [2]. This work provided a new method to synthesize high-performance nanomaterials from MOFs for the construction of electrochemical sensors. Li et al. synthesized a TpPa-1 COF with abundant nitrogen and oxo-functionalities by using 1,3,5-triformylphloroglucinol and phenylenediamine to develop electrochemical sensors and electrocatalysts. Pd^2+^ was employed to modify the TpPa-1 COF by taking advantage of the high adsorption affinity of TpPa-1 COF to Pd^2+^. The Pd^2+^-modified TpPa-1 COF exhibited high catalytic activity for the electrochemical oxidation of hydrazine and the reduction of nitrophenol. The nitrogen-doped carbon derived from TpPa-1 showed superior electrocatalytic activity for the oxidation of reduced glutathione (GSH), which can be used for GSH detection [3]. The synthesized TpPa-1 COFs can be suitable substrates and precursors for potential applications of electrochemical sensing and electrocatalysis.

Carbon nanomaterials, particularly carbon nanotubes and graphene, are among the most investigated nanomaterials for the fabrication of electrodes. Angizi et al. estimated the possibility to develop a pH-sensing platform with several graphene derivatives, and demonstrated the dependence of pH-sensing behaviors on the type and defect density of graphene derivatives. Eventually, a highly pH-sensitive platform was successfully fabricated with thermally annealed graphene oxide, which allowed for a maximum current change of 175% in a pH range from 3 to 9 [4]. Their research could offer a new way to develop micro- and nano-sized pH sensors based on graphene derivatives. Sun et al. designed a portable *E. coli* detection system using the combination of immunomagnetic separation technology, a graphene-based transparent electrode, and the improved adenosine triphosphate bioluminescence technology. Such a system offered a detection range of 3.1 × 10^1^–10^6^ CFU/mL and a coefficient of variation of 3.96%, and no more than 30 min was required to complete the detection. Moreover, it allowed a high accuracy of more than 94% for *E. coli* detection in beverages and food [5]. Stanojev et al. explored the fabrication of a transparent electrode with single-walled carbon nanotubes (SWCNTs) using a combination of experimental tests and computational studies. The density functional tight-binding method was used to explore the interaction of carboxylic SWCNT with some solvents. PEI and carboxylic SWCNT were deposited on soda lime glass substrates to fabricate the thin film electrode, and its stability was tested in different solvents. By optimizing the number of depositions, transparency in the MIR range and conductivity, the electrode with suitable thickness of film enables a reduction of 20% in the MIR’s transparency compared with that of the thickest SWCNT layers, while its sheet resistance can be decreased to 150–200 kW/sq [6].

Graphene oxide shows some unique advantages for electrochemical sensing. Yuan et al. constructed a micro-electrochemical sensor with a graphene oxide-modified screen-printed electrode and a polydimethylsiloxane (PDMS) micro-cell for multi-analyte detection. Their experiment demonstrated that the residual oxygen-containing functional species on graphene oxide can enhance the selectivity of sensors for different targets. Meanwhile, their investigation revealed that the attachment of bovine serum albumin can favor the diffusion of probes rather than hinder electron transfer [7].

Furthermore, many nanotechnologies have been applied to fabricate electrodes to enable them with some unique properties. Li et al. fabricated a flexible and stretchable electrode with a PDMS–Ag nanosheet composite. A low resistivity of 4.28 Wm and a low resistance variation in strain in the range of 0–50% was recorded for the resultant electrode, while the electrical conductivity of the electrode remained stable over 1000 cycles. These attracting characteristics were ascribed to the flexibility and stretchability of PDMS and high electrical conductivity of Ag nanosheets. By coupling the experiment with a theoretical model and finite element simulation, the conductive mechanism of the electrode during stretching was analyzed [8]. This work would facilitate the exploration of flexible electrodes in terms of structural design and material selection and promote the development of flexible electronic and wearable devices. Yan et al. constructed an electrochemical immunosensor using an indium tin oxide (ITO) electrode with an amino-rich nanochannels array for the detection of prostate-specific antigens (PSA). The amino-group-containing precursor was employed to grow the vertically ordered mesoporous silica nanochannel film on ITO, and the introduction of surfactant micelles caused aldehyde groups to anchor recognitive antibodies for the assembly of an immuno-recognitive interface. For detection, the specific recognition of PSA by antibody depressed the diffusion of the probe, causing the electrochemical signal to “Turn-off”. Such an immunosensor offered a detection limit of 8.1 pg/mL and was used in the analysis of human serum [9]. The developed immunosensor can be also applied to develop a universal immunosensing platform for tumor marker detection.

In addition to excellent nanomaterials, strategies to assemble the sensing interface at the electrode are also vital for improving the analytical performance of electrochemical sensors. Chang et al. reviewed advances in the in-situ assembly of nanomaterials on the surface of electrodes for efficient signal readout and amplification. Furthermore, the assembly of organic molecules and biomolecules on electrodes were also discussed [10].

Recently, thanks to their unique advantages, electrochemical sensors are being widely used to monitor the important small molecules in life. Ahmad et al. reviewed the recent advances in electrochemical biosensing of H_2_O_2_, and a subdivision of electrochemical sensors was provided by types of nanomaterials used for fabrication. The strengths and weaknesses of the sensors were discussed by the comparison of their analytical performances. They highlighted the advantages of electrochemical sensors for H_2_O_2_ detection and the vital role of nanomaterials for such sensors in the early detection of cancerous cells [11].

This Special Issue has highlighted the important role of nanomaterials in fabricating high-performance electrochemical sensors. Meanwhile, the results provided by this Special Issue can favor the design and application of electrochemical sensors in the future.
